# Mental Health Policy Name: War and mental health (Croatian experience)

**DOI:** 10.1192/j.eurpsy.2024.67

**Published:** 2024-08-27

**Authors:** M. Rojnic Kuzman

**Affiliations:** Zagreb University Hospital Centre and the Zagreb School of Medicine, Zagreb, Croatia

## Abstract

**Abstract:**

War represents one of the major traumatic events for humans and comes with enormous consequences for individuals and society over a long period of time. War causes acute psychological trauma, but also results in subacute, chronic psychiatric disorders for all those experiencing or witnessing direct war trauma and to those experiencing indirect war trauma resulting from losing the safety of home and financial income, to losing family members and close ones. Therefore, acute reaction to trauma may result in maladaptive disorders and PTSD within days of experiencing trauma and with chronic posttraumatic stress conditions even years after the traumatic experience. Chronic PTSD is associated with higher morbidity of somatic conditions, including hypertension, hyperlipidemia, metabolic syndrome, all resulting in cardiovascular and cerebrovascular disorders. Additionally, according to reports from World Health Organisation (WHO), it has been projected that in emergencies, on average, the percentage of people with a severe mental disorder increases by 1 per cent over and above an estimated baseline of 2–3 per cent. In addition, the percentage of people with mild or moderate mental disorders, including mood and anxiety disorders (including PTSD), may increase by 5–10 per cent above an estimated baseline of 10 per cent. Furthermore, research indicate the possibility of a transgenerational effect of trauma, via maternal psychosocial stress and socioeconomic disadvantage during pregnancy but also through adverse parenting practices, as parenting style may change when exposed to war traumas.

As war affects mental health of different population groups dramatically and long-term, establishment of long term and coordinated mental health care is necessary. In the presentation, examples of practices from Croatia will be discussed.

**Disclosure of Interest:**

None Declared

